# Analysis of the association between salivary proteins and oral mucositis in patients with head and neck cancer undergoing IMRT: a longitudinal study

**DOI:** 10.1186/s12903-024-04400-9

**Published:** 2024-05-29

**Authors:** Maria Gonzalez Agurto, Guy H. Carpenter, Sophie S. Bozorgi, Garrit Koller, Michael Fenlon, Fiona Warburton, Kenneth Bruce, Mary Burke, Avijit Banerjee

**Affiliations:** 1 Faculty of Dentistry, Universidad de los Andes, Santiago, Chile; 2 Faculty of Dental, Salivary Research, Centre for Host-Microbiome Interactions, Oral & Craniofacial Sciences, King’s College London, London, UK; 3 Department of Endodontics, Faculty of Dentistry, Centre for Host Microbiome Interactions, Oral & Craniofacial Sciences, King’s College London, London, UK; 4 Faculty of Dentistry, Oral & Craniofacial Sciences, King’s College London, Guy’s Hospital, Floor 22, London, UK; 5 Faculty of Dental, Oral Clinical Research Unit, Oral & Craniofacial Sciences, King’s College London, London, UK; 6 Institute of Pharmaceutical Science, King’s College London, Franklin-Wilkins Building, King’s College London, London, UK; 7 Guy’s and St Thomas’ NHS Foundation Trust, London, UK; 8 Centre of Oral Clinical Translational Sciences, Faculty of Dentistry, Oral & Craniofacial Sciences, King’s College London, Conservative & MI Dentistry, London, UK

**Keywords:** Head and neck cancer, Oral mucositis, Radiotherapy, IMRT, Salivary proteins, Salivary profile, Radiotherapy side effects

## Abstract

**Introduction:**

This longitudinal study assessed the association between salivary protein composition and the clinical onset/severity of oral mucositis (OM) in patients with head and neck tumours treated with intensity-modulated-radiotherapy (IMRT).

**Methods:**

Saliva samples/clinical data were obtained from 40 head and neck cancer patients treated at Guy’s Hospital before -IMRT(T0) and after-IMRT (T1 = 6 m, T2 = 12 m) (ethics approval/consent).

Salivary flow rate, total protein concentration, and secretion rate were determined from saliva samples and compared with pre-treatment values. OM was assessed, total/specific salivary proteins, including mucin 5B and 7, IgA, cystatin-S, albumin, and α-amylase, were quantified.

**Results:**

95% patients experienced OM during IMRT, with 33 subjects reaching grade 2&3. At T1, there was a significant reduction in salivary flow rate, total protein secretion rate, α-amylase and cystatin-S compared to baseline. Remarkably IMRT did not significantly alter mucin 5B and 7, or the IgA secretion rate at any time point. At T1, all the analyzed proteins were associated with the OM outcomes. In addition, there was a significant inverse correlation between IgA concentration at T0 and the severity of OM during IMRT.

**Conclusion:**

This study revealed significant associations between several salivary proteins and OM in patients with head and neck cancer undergoing IMRT. Further longitudinal studies are needed to confirm these results.

**Clinical significance:**

The study contributes to the understanding of certain salivary proteins association with OM. This could be the first step towards identifying potential salivary markers that could offer perspectives for personalized medicine approaches to improve their quality of life (QoL).

**Research question:**

What is the association between salivary proteins and the occurrence and severity of OM in head and neck cancer patients?

**Aim:**

To assess the association between salivary protein composition with the clinical onset/severity of oral mucositis (OM) in head and neck cancer patients treated with intensity modulated radiotherapy.

**Null hypothesis:**

There is no association between salivary proteins and onset/severity of OM in HNC patients.

**Supplementary Information:**

The online version contains supplementary material available at 10.1186/s12903-024-04400-9.

## Introduction

Head and neck cancer (HNC) ranks as the sixth most common cancer type worldwide, representing 4%–5% of all malignancies. In the United Kingdom, approximately 12,000 people are diagnosed with HNC annually [1–3]. Radiotherapy (RT), often representing the underpinning HNC treatment, is associated with debilitating side effects, oral mucositis (OM) being one of the most common. OM is a painful and debilitating acute oral condition with an incidence of 60%–85%, dramatically deteriorating the quality of life (QoL) and care provision [3, 4].

OM affects cancer treatment schedules through recurrent RT interruptions, complete discontinuation, and dose modification, thereby diminishing the overall treatment efficacy, prolonging recovery, and significantly impacting the QoL. These interruptions reduce therapy compliance, thereby extending the treatment course and compromising its efficacy as well as further compounding poorer outcomes [5–7]. Severe OM could lead to additional medical attention to treatment-related emergency admissions, hospitalization, special diets, parenteral feeding (nasogastric tubes), and palliative symptom management comprising systemic analgesia (opiates) for pain control as well as increased risks of secondary infections, all combining to lower patients’ general well-being during their treatment [5–7].

Beyond the health-related negative effect on the QoL of the patients (impairing physical, emotional, and psychological functional aspects), OM financially impacts healthcare providers, significantly increasing the treatment cost (reaching up to 30,000 USD) per patient, associated only with OM-specific side effects [8, 9].

Nowadays OM treatment is focused on palliative care to alleviate the symptoms, especially the early stages of this side effect. Treatment includes topic and systemic analgesia, mouthrinses, cryotherapy, and photo biomodulation [10].

Intensity-modulated radiotherapy (IMRT) held the promise of diminishing adverse outcomes [10, 11]. IMRT offers higher precision in curative radiation dose delivery (multiple beams) with different intensities and dose escalation, improving target conformity and enabling partial sparing of healthy tissues, particularly that of the salivary glands [5, 6, 8, 12].

Unstimulated whole-mouth saliva (UWMS) constantly flows in the oral environment and contains a mixed secretion. Salivary secretion rate and protein composition could reflect general health and oral status providing information on systemic and oral diseases [13, 14]. Saliva flows over hard and soft oral tissues, moistening and lubricating them, while the salivary protein components are responsible for various functions, including oral mucosal defense [13], forming a protective barrier that maintains oral surface resistance to damage, and promoting microbial diversity and bacterial clearance [15]. Unstimulated whole-mouth saliva collection is minimally invasive, accessible, and easy to perform [16, 17].

Concerning the mucosal barrier layer, its main components are mucin 5B and 7 (high-molecular-weight glycoproteins) as well as secretory IgA (SIgA), the main and most abundant salivary antibody [18, 19]. Mucin 5B is the primary gel-forming mucin in the oral cavity while mucin 7 binds IgA to increase its concentration in the oral mucosa, forming the oral mucosal pellicle [15]. IgA plays an antimicrobial role in the saliva, with commensal bacteria contributing to biofilm formation and preventing pathogen adherence, colonization, and invasion [13, 15, 20]. The altered levels of these proteins could reflect disrupted mucosal defense mechanisms, potentially contributing to OM severity.

α-amylase is the single most abundant protein in the saliva, mostly secreted by parotid acinar cells, with the core function of converting non-soluble polysaccharides into soluble molecules [8, 13, 14, 21]. Cystatin S is mostly produced by submandibular acinar cells, and it could selectively bind to bacterial colonies in the oral cavity, inhibiting bacterial cysteine proteases [13]. Moreover, cystatin S is pivotal for tooth remineralization through binding hydroxyapatite and calcium, thereby inhibiting calcium phosphate salt precipitation and maintaining dental structure [14, 18, 19, 22]. Albumin is not synthesized by the salivary glands, and it is a biomarker for plasma leakage through gingival crevices or within the glandular parenchyma [23].

Conventional RT [24] or IMRT [25] reportedly changes the SFR and biochemical saliva composition. These alterations could hypothetically impair oral tissue hydration and lubrication, becoming a risk factor for OM [4, 6, 13]. The link between salivary proteins and OM remains elusive. Therefore, in this study, we analyzed the relationship between salivary proteins and OM onset and severity in patients with HNC undergoing IMRT. Understanding these mechanisms would represent the first step toward improving treatment experience, adherence, and survival along with HNC patient QoL.

## Methods

### Participants

Over a one-year period (February 2017–February 2018), we recruited a cohort of 40 patients diagnosed with HNC at the Special Care Dentistry Unit of Guy’s and St Thomas’ NHS Foundation Trust, London, UK, prior to IMRT initiation. We evaluated all patients before, six months after, and 12 months after IMRT (T0, 1, and 2, respectively). All participants were clinically examined by the same consultant (clinical lead for dental oncology service) at all time points at the above-mentioned special dental care unit. In addition, a consultant from the Guy’s and St Thomas’ NHS Foundation Trust (GSTT) oncology team assessed all patients during and after IMRT (2 weeks, 6 weeks, 3 months, 6 months, and 12 months post IMRT).

Eligible HNC patients comprised volunteers aged over 18 years with permanent dentition and without distant metastasis. The exclusion criteria were as follows: systemic diseases that affect the salivary flow rate (SFR) and antibiotic administration in the preceding three months of the study. As an IMRT regimen, we used the national standard of care in the UK at the time of the trial design (i.e., 65–70 Gy with 2.2 Gy daily, chemotherapy of 75 mg/m^2^ up to 3–4 cycles, drugs used were cisplatin/carboplatin). The oncology team defined the protocol for target volume definition, treatment delivery, radiation dose, and fractioning at GSTT following the UK guidelines to preserve the health of the exposed tissues [26].

### Saliva collection and assessment

We collected the UWMS samples at three time points: at the baseline, i.e., before IMRT initiation (pre-IMRT, T0), 6 months post-IMRT (± 1 month, T1), and one-year post-IMRT (± 1 month, T2). Moreover, at the three clinical evaluation time points during the study, the same dental specialist examined clinically the participating patients.

We collected the UWMS samples using the passive drooling method over a 10-min period, following 60 min of fasting. We collected the samples during the day, regularly between 13:30–15:30 h, to eliminate potential circadian physiological variation effects. We transported the samples to the laboratory, aliquoted and centrifuged them (at 10,000 g and 4 °C for 5 min), then stored the aliquots at − 80 °C. We calculated the SFR to measure the secreted salivary volume per minute (mL/min).

### Total protein concentration (TPC) analysis

We assessed the total protein content of the samples using a commercial bicinchoninic acid (BCA) protein assay kit (Thermo-Fisher Scientific, IL, USA) and bovine serum albumin (BSA) as a standard. We diluted the samples at a ratio of 1:10 in ultra-high-quality water and analyzed them in duplicates. We incubated the plates at 37 °C for 30 min, then measured the absorbance at 540 nm with a plate reader (iMark Microplate Absorbance reader BIO-RAD, UK).

### Total protein secretion rate (TPS)

We calculated the total protein secretion rate by multiplying the SFR (mL/min) and protein concentration (µg/mL) to obtain µg/min.

### Protein analysis

#### Mucin 5B and 7 analysis

Following total protein determination, we performed mucin 5B and 7 analysis using SDS-PAGE gel electrophoresis. We adjusted equal amounts of salivary proteins to 20 g and loaded on NuPAGE Novex, 4%–12% bis–tris gels under reducing conditions and heat. We separated the proteins present in the saliva samples by molecular weight. Next, we stained mucin 5B and 7 with polysaccharide periodic acid–Schiff reagent to identify mucin glycosylation. We used Coomassie Brilliant Blue R250 (Sigma-Aldrich, Gillingham, UK) to visualize the overall protein profile in the saliva samples. We measured mucins by comparing the band densitometry of the sample-loaded gels against a linear equation from a standard curve generated based on serially diluted mucin standards [27]. We scanned the sample-loaded gels using an automated image-developing system, ChemiDoc MP Imaging System (Bio-Rad, Hemel Hempstead, UK), then analyzed the gels using the ChemiDoc Complementary Software ImageLab (version 6.0 build 16; Bio-Rad, Hemel Hempstead, UK) in duplicates. Both mucins standard curves and detailed method are in the annexes.

#### α-amylase analysis by kinetic assay

We investigated the α-amylase enzymatic activity in the saliva samples using a commercial α-amylase kinetic assay (Sialimetrics LLC, PA, USA). We diluted the saliva samples using α-amylase diluent (1:200) and compared them with a standard upon the addition of α-amylase substrate (heated to 37 °C) to each well. We measured the absorbance at 405 nm and two time points (i.e., 1 and 2 min) in a plate reader (iMark Microplate Absorbance reader BIO-RAD, UK). We analyzed the samples in duplicates.

#### Albumin analysis by sandwich enzyme-linked immunosorbent assay (ELISA)

We coated the ELISA plates overnight using an albumin capture antibody (Duo-Set Elisa R&D Systems, Minneapolis, USA), then washed them thrice in phosphate-buffered saline Tween (PBS-T). We blocked the ELISA plates with 1% BSA in PBS (pH 7.2) for 1 h, followed by three further PBS-T washes. We diluted the samples in duplicates along with the standard and incubated them at room temperature and pressure for 2 h followed by three PBS-T washes. We supplemented the samples with a biotinylated mouse antihuman serum albumin detection antibody diluted with 1% BSA in PBS and incubated them at room temperature for 2 h followed by three PBS-T washes. We diluted horseradish peroxidase-conjugated streptavidin with 1% BSA in PBS and added it to the samples at room temperature and pressure, then washed them thrice with PBS-T. We added substrate solution (tetramethylbenzidine) in the wells and stopped the reaction with 2 M sulfuric acid after 5 min. Finally, we read the plates at 400 nm using a plate reader (iMark Microplate Absorbance reader BIO-RAD, UK). We analyzed the samples in duplicates.

#### Cystatin S analysis using sandwich ELISA

In this study, we used Cystatin S (CST4, Sandwich Cloud Clone Corp., USA), precoated with a specific antihuman polyclonal antibody to cystatin S. We diluted the samples (1:500 in ultra-high-quality water) in duplicates along with the standard and incubated them at 37 °C for 1 h, then removed the liquid from each well without washing. We diluted in the assay diluent a biotinylated mouse antihuman serum cystatin S detection antibody, added it to the samples, incubated them at 37 °C for 1 h, then washed them thrice with wash solution diluted in ultra-high-quality water(1:30). We added horseradish peroxidase-conjugated streptavidin to the samples for 30 min at 37 °C, followed by five final washes. We supplemented the samples with a substrate solution consisting of H_2_O_2_ and tetramethylbenzidine (1:1) and incubated them for 20 min at 37 °C. We terminated the reaction using 2 M sulfuric acid and read the plates at 450 nm using a plate reader (iMark Microplate Absorbance reader BIO-RAD, UK).

#### IgA analysis by sandwich ELISA

In this study, we used a pre coated 96 wells commercial IgA sandwich kit (Cusabio Biotech USA). We diluted the saliva samples (1:100) in ultra-high-quality water in duplicates along with the standard and incubated them at 37 °C for 2 h, then removed the liquid from each well without washing. We added a biotinylated mouse antihuman IgA detection antibody in the wells and incubated the samples at 37 °C for 1 h, followed by three washes with wash buffer diluted (1:25 in ultra-high-quality water). Next, we added horseradish peroxidase-conjugated streptavidin in the wells and incubated the samples for 1 h at 37 °C, followed by five washes. We supplemented the samples with a substrate solution consisting of H_2_O_2_ and tetramethylbenzidine (1:1) and incubated them for 20 min at 37 °C. We terminated the reaction with 2 M sulfuric acid and read the plates at 540 nm using a plate reader (iMark Microplate Absorbance reader BIO-RAD, UK).

In this study, we assessed IgA using ELISA following the Proctor and Carpenter protocol [25, 28, 29] for saliva analysis, allowing for the detection of the total IgA in the saliva samples, including that of SIgA. Regarding SIgA, this antibody reportedly contains an additional 80-kDa glycoprotein, referred to as the secretory part, which binds the polymeric Ig receptor (plgR) on the epithelial cells, to be transported to the mouth [30, 31].

### Clinical assessment of oral mucositis

A consultant from the GSTT oncology team performed the clinical assessment of OM during and after IMRT (2 weeks, 6 weeks, 3 months, 6 months, and 12 months) using the World Health Organisation (WHO) OM scale, based on the clinical examination of the oral cavity, combining signs of erythema and ulcers with the ability of the patients to eat, in order to assess functionality (WHO, Handbook, 1979). The scoring scale was as follows: grade 0, no OM; grade 1, erythema and soreness; grade 2, presence of ulcer(s), but the patient can swallow solid food; grade 3, presence of painful ulcer(s), the patient was unable to eat; grade 4: presence of ulcer(s), impeding any oral alimentation.

### Statistical analysis

A study with an effect size of 0.5 and 80% power to detect the true difference in all parameters before and after the cancer treatment would require a total sample number of 35 patients, analyzed using a two-tailed t-test with a 5% significance level. We performed the power calculation using Gpower 3.1.5. software (Franz Faul, Universitat Kiel, Germany). We tested the data for normality and analyzed it using the Wilcoxon matched-pair test for testing longitudinal differences within the same patient. We analyzed the data from different OM severity groups using Mann Whitney test and Kruskal Wallis test with Dunn correction to compare the differences between independent groups. We used random effects linear regression in a longitudinal panel to analyze the data obtained from each patient over time to determine the association between the tested proteins and clinical outcomes. We used a logistic regression model to determine the association between the protein data obtained pre-IMRT (independent variable) and the OM onset (0–1) as well as the severity (0–3) during IMRT (dependent variable). We performed all analyses using STATA 15.1 (College Station, Texas USA), GraphPad Prism 8 software (La Jolla California USA), and Microsoft Excel 2018. We considered P-values of *p* < 0.05 statistically significant.

## Results

Table 1 summarizes the baseline patient demographic and tumor characteristics along with the treatment details and health-related lifestyle factors. Radiation dose (mean: 62.5 Gy) along with the fractioning plan (36 patients received 30 fractions of 2.2 daily), were similar (*p* > 0.05). There was no difference regarding bilateral dose, and chemotherapy (CHT), 25 received cisplatin (*p* =  > 0.99), and tumor stages.

**Table 1:** Demographics and clinical characteristics of the HNC patients. Dx represents diagnostic time. Data is expressed as mean ± S.D, percentages

**Patients Recruited**	40
**Age** (Years)	62.5 (SD 13)
Range	44—75
Gender	
Male	36 (90%)
Female	4 (10%)
**Social history**	
** Risk factors**	
Smoking (at Dx)	Yes 27 (70%)
	No 9 (20%)
	Unknown 3 (10%)
Mean day	12.5 cigarettes
Alcohol (at Dx)	Yes 25(55%)
	No 12(22.5%)
	Unknown 3(22.5%)
Mean weekly	11 units
HPV status (at Dx)	Yes 10 (25%)
	No 28 (70%)
	Awaited Dx 2 (5%)
HPV Positive Tumour location	Tonsil 7
	Oropharynx 1
	Tongue 1
	Neck1
**Primary Tumour histology**	Number of patients (%)
Squamous cell carcinoma	38 (95%)
Adenocarcinoma	1 (2.5%)
Unknown	1 (2.5%)
**Tumour stage (TNM)**	Number of patients (%)
**T**	T0 1 (2.5%) T1 3 (7.5%) T2 16 (40%) T3 5 (12.5%) T4 13 (32.5%) 2 unknowns
**N**	N0 11 (27.5%) N1 4 (10%) N2 22 (55%) N3 1(2.5%) N4 0 (0%) 2 unknowns
**M**	M0 38 (95%) 2 unknowns
**Patients’ treatment**	
IMRT + Chemotherapy	25 (57.5%) CISPLATIN (2 doses)
	2 (5%) CARBOPLATIN (2 doses)
IMRT + Surgery	3 (7.5%)
30 Fractions	36 (90%)
Bilateral dose	19 (47.5%)
Unilateral dose	20 (%)
Mean dose	62.5 Gy (20–71)
Median dose	65 Gy
Fractioning regime	2.2 daily
**Tumour Location**	Number of patients (%)
Tonsil	12 (30%)
Oropharynx	6 (15%)
Tongue	5 (12.5%)
Nasopharynx	2 (5%)
Hypopharynx	3 (7.5%)
Larynx	3 (7.5%)
Neck	2 (5%)
Epiglottis	2 (5%)
Parotid	1 (2.5%)
Nasal	1(2.5%)
Mandibular	1(2.5%)
Buccal mucosa	1(2.5%)
Sinus	1 (2.5%)
**Medication**	Number of patients (%)
Not reported	7 (17.5%)
Bisphosphonates	4 (10%)
Immunosuppressive agent	6 (15%)
Steroid	9 (22.5%)
Antihypertensive	13 (32.5%)
No medication	5 (12.5%)

*Dx* Diagnostic, *G* Grey, *TNM* system T extent of the tumor, *N* extent of spread to the lymph nodes, and *M* presence of metastasis

### Salivary flow rate variation at three time points

The patients exhibited a statistically significant reduction in the UWMS flow rate at T1 and T2 (*p* < 0.0001; mean: 0.16; SD: 0.02 mL/min and *p* = 0.001; mean: 0.24; SD: 0.03 mL/min, respectively) compared with that at T0 (mean: 0.44; SD: 0.04). The IMRT bilateral dose did not exhibit any difference in the SFR (*p* = 0.35) at the baseline and T2 (*p* = 0.155).

The sensitivity analysis focusing on salivary flow rate and composition of patients taking antihypertensive medication revealed no statistical difference between T0, T1, and T2.

### Total protein concentration (TPC) and total protein secretion rate (TPS) variation

**Fig. 1:**
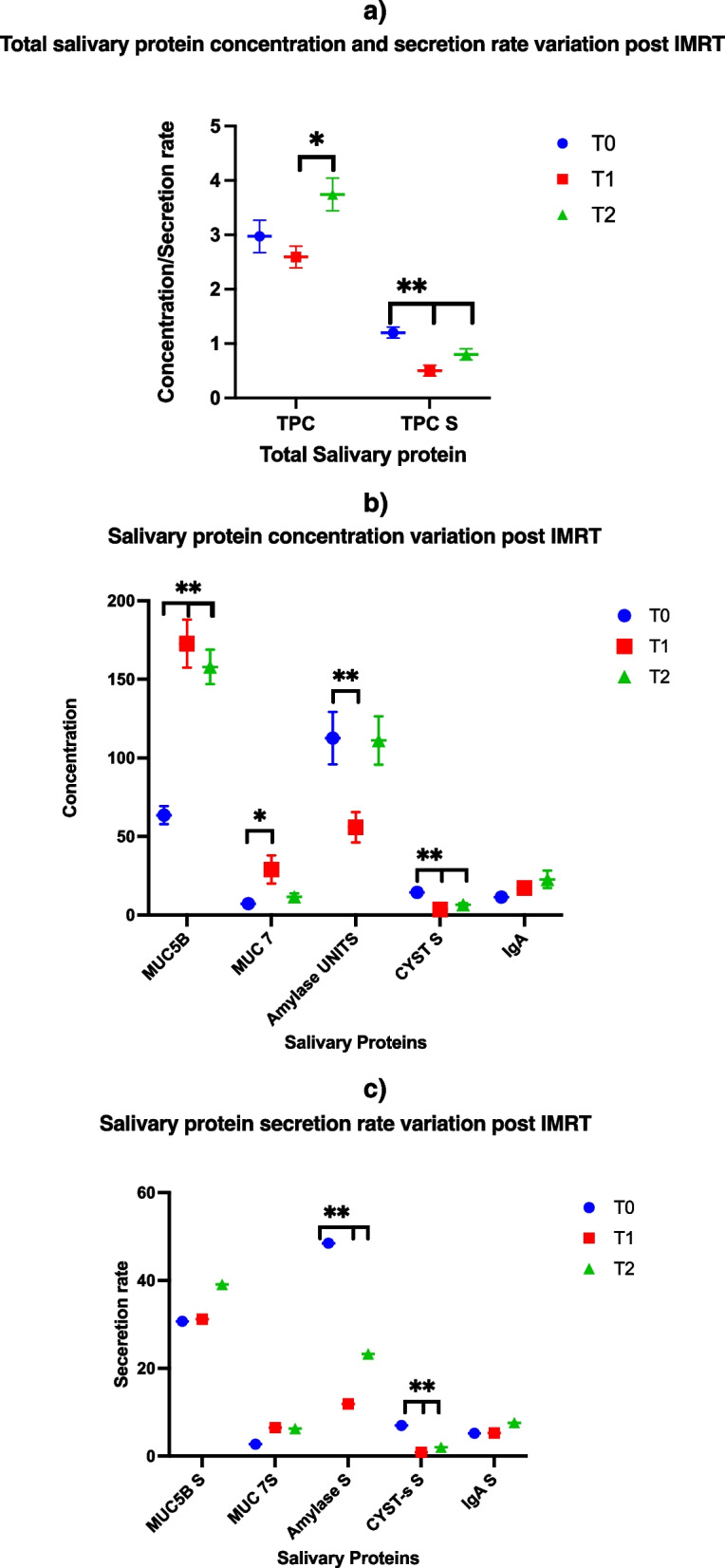
Protein concentration and secretion rate variation post-IMRT at T1 and T2 compared with baseline T0. **a** TPC represents total protein concentration, TPS represents total protein secretion rate, **b**) MUC mucin, Amy α-Amylase, Alb albumin, CST4 cystatin s, IgA immunoglobulin A concentration at T0, T1, T2. **c** MUC mucin, Amy α-Amylase, Alb albumin, CST4 cystatin s, IgA immunoglobulin A secretion rate variation at T0, T1, T2. In **a**, **b** and **c** superscripts, * represents significant *p*-value differences < 0.05 and b < 0.01 and ** represents significant *p*-value differences < 0.001. post IMRT. Total protein concentration (mg/ml), selected protein concentration(ug/ml), amylase (U/ml). T0 before IMRT, T1 6 months post-IMRT, T2 12 months post-IMRT

Next, we assessed the total protein concentration and secretion rate variation before (T0) and after IMRT (T1 and T2). The TPC increased by T2, potentially linked to a reduced post-IMRT salivary volume. We observed a significant reduction in the post-IMRT total protein secretion rate compared with that at the baseline. At T2, this slightly recovered compared with that at T1 without reaching the pre-IMRT value (Fig. 1a).

Following a similar pattern, when comparing unilateral and bilateral radiation dose related TPC and secretion rate values, we observed no statistically significant differences at any time point: i.e., TPC T0 (*p* = 0.98), T1 (*p* = 0.32), and T2 (*p* = 0.7); TPS T0 (*p* = 0.687), T1 (*p* = 0.244), and T2 (*p* = 0.13).

### Specific salivary protein concentrations and secretion variations

Figure 1b and Fig. 1c summarize every protein concentration and secretion rate we assessed at T1 and T2 compared with those at the baseline (T0). Furthermore, this figure highlights the protein concentration- and secretion rate-related variations. We detected significantly increased mucin 5B (*p* =  < 0.001) and 7 concentrations (*p* =  < 0.01), probably related to the reduced SFR that affected this patient group. The post-IMRT secretion rate of both mucins was not statistically different (*p* =  > 0.05). We registered reduced α-amylase S (*p* =  < 0.01), albumin S(*p* =  < 0.01), and cystatin S S(*P* =  < 0.001) at T1-T2 compared with those at T0. The IgA levels did not change significantly between the three time points (*P* =  < 0.05). However, the post-IMRT IgA concentration and secretion rate increased.

### OM assessment during IMRT

Our OM assessment revealed that 80% of the investigated patient group presented side effects during the cancer treatment. Table 2 summarizes the side effect presence and severity in the patient cohort at different time points along with the maximum severity reached during IMRT. Moreover, we observed no difference in OM onset (*p* = 0.45) and maximum severity of mucositis (grades 2 and 3; *p* = 0.94) concerning IMRT and IMRT-chemotherapy.

**Table 2:** Oral mucositis onset and severity at different time points

characteristic	Oral Mucositis Presence	Oral Mucositis Grade		
	**Yes (n)**	**Grade 1 (n)**	**Grade 2 (n)**	**Grade 3 (n)**
**Time**				
During IMRT	37(95%)	4 (12.5%)	22 (40%)	11 (27.5%)
Post IMRT				
T1	6(16%)	6 (15.7%)	-	-
T2	3(9%)	3 (9%)	-	-
**IMRT dose**				
Unilateral	19 (47.5%)	1.9 ± 0.8522		
Bilateral	17 (42.5%)	2.2 ± 0.6468		

mean ± SD, T1 6 months post IMRT, T2 12 months post IMRT, unilateral and bilateral IMRT dose received by each participant

n = number of participants (%)

Our OM onset/grade evaluation during IMRT between patients who received unilateral and bilateral RT doses did not yield any difference (*p* = 0.6 and *p* = 0.2548, respectively). Finally, we could not detect any difference in the OM onset between smokers and nonsmokers (*p* = 1) or that related to alcohol consumption (*p* = 0.5).

### Association between OM outcomes and salivary biochemical components

#### Post-IMRT salivary protein concentration and secretion rate and association with OM onset

Next, we used random effects linear regression in a longitudinal panel to investigate the relationship between the aforementioned salivary proteins and the OM clinical parameters in order to reveal their potential clinical and biological association. Table 3 presents the statistically significant association between the analyzed proteins and the T1 post-IMRT OM, which were all positive. In addition, the longitudinal analysis of the OM onset and severity outcome measures and saliva flow rate revealed a negative and significant association between OM and SFR at T1 and T2 (*p* = 0.0001). Furthermore, we described positive and significant associations between OM and both total protein concentration at T1 (*p* = 0.009) and secretion rate at T1 (*p* = 0.01). We observed no statistically significant associations post-IMRT between OM and mucin 5B and 7 concentrations. In contrast, mucin 5B and 7 secertion rate s displayed statistically positive and significant associations at T1 (*p* = 0.01 in both cases). Similarly, the α-amylase units and secretion rate were both statistically significantly (positive) associated with OM presence at T1 (*p* = 0.04 and *p* = 0.03, respectively). Similarly, albumin concentration and secretion rate were both significantly associated with OM at T1 (*p* = 0.007 and *p* = 0.01, respectively). In contrast, we observed no statistically significant associations at T2. The cystatin S concentration was significantly and positively associated with OM at T1 (*p* = 0.03) and its secretion rate appeared to have a borderline association at T1 (*p* = 0.05) with OM. Importantly, solely the IgA concentration displayed a significant association with OM both at T1 and T2 (*p* = 0.007 and *p* = 0.03, respectively). IgA secretion rate was significantly associated with OM at T1 (*p* = 0.009) but weakly with that at T2 (*p* = 0.05).

**Table 3:** Statistically associations over time between oral mucositis onset and tested salivary proteins (Random effects linear regression in a longitudinal panel)

Clinical outcome and proteins		Coefficient	*P* value	[95% Conf.	Interval]
**OM/TPC**	T1	**0.153**	**0.009**	0.038	0.267
**OM/TP S**	T1	0.156	**0.01**	0.0281	0.283
**OM/MUC5B S**	T1	0.138	**0.01**	0.0247	0.252
**OM/ MUC7 S**	T1	0.135	**0.01**	0.026	0.244
**OM /α-AMYLASE U**	T1	0.107	**0.04**	0.004	0.208
**OM/α-AMYLASE U S**	T1	0.118	**0.037**	0.007	0.229
**OM/ALBUMIN**	T1	0.151	**0.007**	0.040	0.262
**OM/ALBUMIN S**	T1	0.136	**0.01**	0.027	0.245
**OM/Cystatin-s**	T1	0.171	**0.03**	0.008	0.334
**OM/Cystatin-s S**	T1	0.143	**0.05**	-0.004	0.291
**OM/IgA**	T1	0.156	**0.007**	0.041	0.271
	T2	0.140	**0.03**	0.013	0268
**OM/IgA S**	T1	0.154	**0.009**	0.039	0.269
	T2	0.126	**0.05**	-0.001	0.254

T1 time 16 months post IMRT

T2 12 months post IMRT

*SFR* Salivary flow rate, *TPC* Total protein concentration, *TP* Total protein, *C* Concentration, *S* Secretion rate, *U* Units, *MUC 5B* Mucin 5b, *MUC7* Mucin 7

### IgA assessment

#### Pre-IMRT-IgA concentration and secretion rate analysis of OM severity outcomes

We discovered that both the IgA concentration and secretion rate were significantly associated with OM both at T1 and T2. Therefore, we analyzed the pre-IMRT IgA concentration and secretion rate on a subgroup regarding different OM severities.

Concerning the pre-IMRT IgA concentration analysis among different OM severity (grades 0,1,2, and 3), we divided OM severity during IMRT into two groups, i.e., grades 2 and 3 (*n* = 33) vs 0 and 1 (*n* = 7)when comparing the pre-IMRT IgA concentration from patients reaching grades 2 and 3 (mean: 10,36; SD ± 5,065) with those at grades 0 and 1 (mean: 17,72; SD ± 4,362) during IMRT and observed a significant difference (*p* = 0.0073). Afterwards we divided into 3 groups of patients presenting different OM severity grades as it follows 0/1, 2, and 3. Among these 3 groups the pre-IMRT-IgA concentration was significantly different (*p* = 0.02). In addition, Dunn correction was applied showing significant differences between OM grades 0/1 (*n* = 7) and grade 2 (*n* = 22) (*p* = 0,02), as well between OM grades 0/1 (*n* = 6) and 3(*n* = 11) (*p* = 0.046).

#### Pre-IMRT IgA concentration and secretion rate association with OM severity during IMRT

Finally, we assessed the association between clinical outcomes regarding OM severity during IMRT and pre-IMRT (T0) salivary biochemical composition. In this context, we analyzed the possible relationship between salivary IgA concentration at T0 and OM severity during the cancer treatment. OM grades 2 and 3 during radiotherapy were inversely and significantly associated with the pre-IMRT IgA concentration at T0 (*p* = 0.017). In addition, an increase in IgA protein concentration at T0 would be associated with a 20% (95% CI: 4%–33.5%) of lower chance of develop OM grades 2 and 3 during IMRT.

## Discussion

In our study, as expected, we revealed that IMRT significantly and detrimentally affected the salivary glands, reducing flow rate and total protein to a third of the pre-IMRT levels even after 6 months, although both values recovered to a certain extent by 12 months. Significantly, IMRT did not affect salivary mucin or IgA levels at either time point. These proteins are crucial for the lubrication and immunological defense of the oral mucosa [15]. Although we collected no saliva samples either during or immediately after IMRT, we assumed that the observed salivary inhibition pattern would be present during IMRT based on the literature [32]. Nearly all patients receiving IMRT experienced OM during IMRT, which persisted in certain patients even at 6 months. This result suggests that IMRT-induced OM is unrelated to the loss of the lubricating components of the saliva but it is rather associated with the direct effect of IMRT on the epithelial cells (e.g., DNA strand breaking, triggering oxidative stress reactive oxygen species generation in sub epithelial tissue, activating and inducing innate immune response) as well as subsequent complex inflammatory chain response, activating and inducing an innate immune response. However, it could be related to other factors such as oral microbiome dysbiosis [33] or oral mucosa binding protein (mucin 1) alterations, which would impair pellicle formation, thereby altering the protective functions regarding lubrication, hydration, protection against degrading enzymes, microbial invasions as well as the defense against infections and mucosal diseases. Moreover, altered mucin 1 expression could lead to mucosal irritation, erosion, ulcers, and OM [34, 35]. Undoubtedly, the secretory capacity of the major salivary glands was reduced [13] as parotid and submandibular secretory marker (i.e., amylase [36] and cystatin S [37], respectively) levels both significantly decreased at 6 months post-IMRT as well as the salivary flow [38]. However, this reduction was temporary and both glands secretory capacity was recovered to a certain extent at T2. In agreement with other studies [39], the parotid gland appeared to recover faster as the amylase rate returned to the pre-IMRT levels after 12 months whereas that of cystatin did not [37]. Typically, UWMS comprises more submandibular/sublingual than parotid saliva [20, 40]. IMRT did not reduce the IgA secretion rates. In contrast, we observed a trend of increased IgA secretion at T2, similar to that described in other studies that used ELISA techniques to assess IgA [25, 41]. This increased concentration and secretion rate represent the total IgA present in the saliva, including secretory IgA, potentially reflecting increased plasma cell infiltration into the saliva related to damage. However, an increased mucin concentration in a reduced salivary volume might negatively affect the rheological properties of the saliva, increasing viscosity, which would impair its lubricating ability in the oral cavity. Therefore, investigating other factors that could affect oral lubrication would be interesting. The most obvious candidate would be mucin 1, the oral epithelial cell membrane-bound mucin that anchors salivary mucins to the surface [42]. In addition, altered glycan composition could modify much in configurations, resulting in tight-packed globular aggregates with reduced water retention capacity [13, 43]. We observed unchanged albumin secretion rate at T1, surprisingly exhibiting a significant reduction at T2 compared with that at T0, suggesting that the oral side effects of IMRT, did not affect the albumin transfer into the saliva, contrary to the results of previous observations [44, 45]. However, Ventura studied the enamel and mucosal pellicle, in which increased albumin concentrations were reported in 9 post-RT (3–4 months) patients with HNC compared with pre-RT results [45].

Concerning the primary tumor site, most patients presented locations anatomically close to each other and near the parotid and submandibular glands. Therefore, these two salivary glands could be potentially affected by the toxic IMRT side effects [20]. Clinically, this is shown by the reduction in SFR and the changed total protein secretion rate after IMRT.

We revealed a negative and significant association between the pre-IMRT IgA concentration and OM maximum severity during cancer treatment, indicating that patients with lower pre-IMRT IgA saliva concentrations could be more prone to develop grade 2 and 3 OM (grade 2 and 3 mean IgA concentration: 10.4; grade 1 mean IgA concentration: 17.7). In the literature has been stablished that a reduced IgA concentration at the mucosal surfaces would impair host-microbial homeostasis, adherence, and protection from bacterial infection, thereby altering bacterial diversity and biofilm formation [18, 46]. Moreover, IgA is vital for commensal bacterial colonization in gut mucosal tissues [46] and it could bind certain bacteria, facilitating the colonization of the oral mucosa [47] to maintain a healthy and functional mucosal barrier, which is critical during OM onset and development to avoid secondary infection [17]. Therefore, a reduced IgA concentration pre IMRT would impair this interaction, affecting colonization levels, mucosal protection against toxins and infections increasing the risk of infection [48]. This aspect should be further investigated during the IMRT as well as the baseline records (pre-IMRT) of cancer patients with non-HNC patients to better understand this association.

In our study, 37 patients (94%) developed OM during the IMRT, and none displayed severity grade 4; however, 30% of the patients suffered from OM severity grade 3. These findings were similar to those of a previous study [49], describing severe acute OM (grade ≥ 3) in 30% of the IMRT-treated patients with HNC. Equally, another previous study also reached similar conclusions [50], reporting 0% of grade 4 cases in post-IMRT patients with HNC.

We detected significant associations at T1 and T2 between OM and the reduced SFR in all participants. This association alludes to the role of saliva throughout the development of OM and severity concerning mucosal wetting, and lubrication as well as bacterial protective functions and colonization [39, 51]. However, the clinical relevance of such findings depends on the salivary composition-related changes [39]. We revealed significant positive associations between a significantly reduced total protein secretion rate and OM at T1, thereby salivary properties were altered.

OM onset is caused by the direct effect of IMRT on epithelial cells, provoking a complex sequence of physical-biological events that interact during OM development, the most relevant being the presence of extensive, deep, and painful ulcers. In addition, OM could be affected by patient-related factors. These significant associations between the number of patients presenting OM (n = 6) and mucin 5B and 7 secretion rates at T1 might indicate that the protective mucosal layer was changing and no longer helping to maintain mucosal integrity during RT, when the OM cases reached a total of 37, with 11 being grade 3 severity cases. Furthermore, the reduced SFR and the increased mucin 5B and 7 levels affected salivary viscoelasticity, making the saliva “stickier” and more viscous, thereby reducing its functional value, resulting in an impaired protective barrier [52].

In summary, our analysis suggests that IMRT could limit the damage caused to the salivary glands and, importantly, allows for functional recovery. This study provides insight into the implications of salivary lubricating factor loss as a patient-related and OM-associated factor. Furthermore, the clinical importance of this study becomes even more obvious when considering the associations between clinical and biochemical data to identify potential markers for increased susceptibility to severe OM in a larger group of patients with HNC. To date, no therapeutic agents exist to resolve or reduce OM duration, which also remains a dose-limiting factor in HNC treatment. Patients affected by OM tend to interrupt or even terminate their treatment course early as they undergo significant QoL deterioration, despite the adverse effect on their survival outcome [6, 14, 17, 30, 53]. A limitation of OM research is the lack of universal, objective, and standardized indices for data collection, and the outcomes depend mainly on the clinical experience and training of the consulting physician [7, 54]. Nevertheless, the WHO toxicity scale has been used extensively since 1979 [54]. The number of medical appointments, as well as variuos side effects that appear after cancer treatment, could complicate sample collection, thereby reducing study protocol compliance. However, the dropout rate in this study was low (7 patients). The reduced post-IMRT SFR made sample collection and analysis more difficult. Another limitation of this study was the sample size, conclusions should thus be made with caution. Larger-scale longitudinal studies would be required to confirm the outcomes. In addition, a prospective study, including sample collection during IMRT, would be necessary to assess the temporal aspects of OM onset and severity. Finally, the total IgA assessment concentration and secretion rate might be overrepresented as the ELISA antibody would bind both types, including secretory IgA.

## Conclusions

The present study aimed to investigate the association between specific salivary proteins and the occurrence and severity of OM in patients with head and neck tumors. The results suggest that IMRT has no effect on salivary mucins or IgA levels, which play a crucial role in oral mucosal lubrication and immune defence. These results emphasize the role of IgA levels prior to IMRT in the severity of OM in this group of HNC patients. The null hypothesis was rejected, salivary proteins were associated with OM in HNC patients undergoing IMRT. The biochemical and clinical associations served as a first step to analyze pretreatment salivary proteins in patients undergoing IMRT to monitor OM, mainly IgA.

Further longitudinal studies are needed to confirm these results and better understand the underlying mechanisms, also to improve care protocols during treatment.

### Supplementary Information

Supplementary Material 1.

Supplementary Material 2.

## Data Availability

The data was submitted under Research Data Protection Registration at King’s College London research governance: KDPR Registration Reference: DPRF-17/18–6377. Data is provided as a supplementary information files.

## Abbreviations

BSA: Bovine serum albumin
ELISA: Enzyme-linked immunosorbent assay
GSTT: Guy’s and St Thomas’ NHS Foundation Trust
HNC: Head and neck cancer
ROM: Radiation Induced Oral Mucositis
PBS: Phosphate-buffered saline
QoL: Quality of life
SFR: Salivary flow rate
S: Secretion rate
TPC: Total protein concentration
TPS: Total protein secretion rate
UWMS: Unstimulated whole-mouth saliva
WHO: World Health Organisation
CHT: Chemotherapy
