# Supplementary material for: Analysis of the association between salivary proteins and oral mucositis in patients with head and neck cancer undergoing IMRT: a longitudinal study
Source: BMC Oral Health. 2024 May 29;24:630. doi: 10.1186/s12903-024-04400-9 (PMC11134661; doi:10.1186/s12903-024-04400-9)
Supplement: Supplementary file 1 — Supplementary Material 1. [file 12903_2024_4400_MOESM1_ESM.docx]

**Annexes**

#### Mucin Semi-quantification

MUC5B and MUC7 mucin glycoproteins were semi-quantified using purified mucin fractions of known concentrations (Malmo University, Sweden). The purified mucin fractions were serially diluted, underwent electrophoresis and stained with PAS. Images of the PAS stained gel were analysed for band intensity using the ChemiDoc. The bands were converted to peaks and the area under each curve gave the pixel intensity. Standard curves and linear equations were generated for mucins (MUC5b and MUC7) concentration against pixel intensity. The mucin concentrations of the samples were calculated from the known pixel intensities using the linear equation obtained from the standard curve shown in Figure 1.

(A)
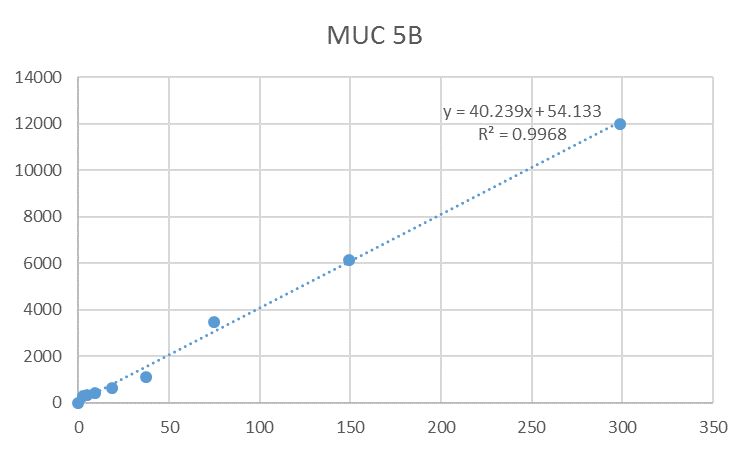


(B)
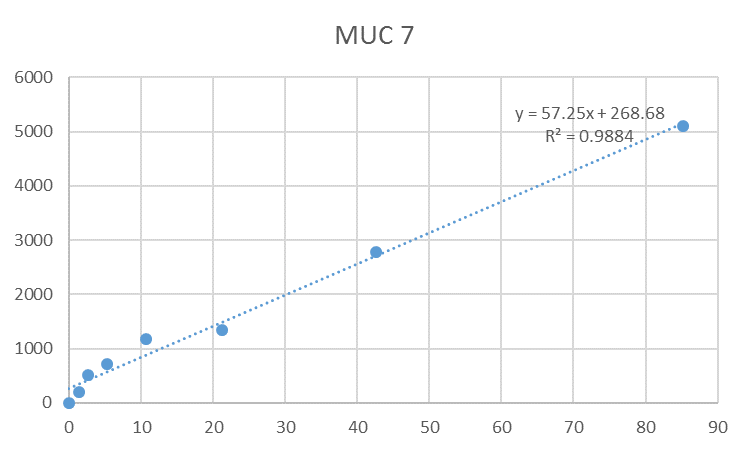


Figure 3.1 Mucin standard-concentration curves for (A) mucin 5B, (B) mucin 7 .

Purified mucins with their linear equations used to calculate the concentration of mucin 5B and mucin 7 in the different test samples.

Reference

1. Gonzalez Agurto, M. (2019). *ORAL EFFECTS OF RADIOTHERAPY IN HEAD AND NECK CANCER PATIENTS; AETIOLOGY AND PATHOPHYSIOLOGY LINKED TO MANAGEMENT*.
